# Crop diversity loss as primary cause of grey partridge and common pheasant decline in Lower Saxony, Germany

**DOI:** 10.1186/s12898-016-0093-9

**Published:** 2016-09-09

**Authors:** Katrin Ronnenberg, Egbert Strauß, Ursula Siebert

**Affiliations:** 1 Institute for Terrestrial and Aquatic Wildlife Research, University of Veterinary Medicine Hannover, Foundation , Bischofsholer Damm 15, 30173 Hannover, Germany; 2 Hunting Association of Lower Saxony, Schopenhauerstraße 21, 30625 Hannover, Germany

**Keywords:** Habitat model, Trend analysis, Grey partridge, Common pheasant, Citizen science, Diversity, Maize cultivation

## Abstract

**Background:**

The grey partridge (*Perdix perdix*) and the common pheasant (*Phasianus colchicus*) are galliform birds typical of arable lands in Central Europe and exhibit a partly dramatic negative population trend. In order to understand general habitat preferences we modelled grey partridge and common pheasant densities over the entire range of Lower Saxony. Spatially explicit developments in bird densities were modelled using spatially explicit trends of crop cultivation. Pheasant and grey partridge densities counted annually by over 8000 hunting district holders over 10 years in a range of 3.7 Mio ha constitute a unique dataset (wildlife survey of Lower Saxony). Data on main landscape groups, functional groups of agricultural crops (consisting of 9.5 million fields compiled by the Integrated Administration and Control System) and landscape features were aggregated to 420 municipalities. To model linear 8 or 10 year population trends (for common pheasant and grey partridge respectively) we use rho correlation coefficients of densities, but also rho coefficients of agricultural crops.

**Results:**

All models confirm a dramatic decline in population densities. The habitat model for the grey partridge shows avoidance of municipalities with a high proportion of woodland and water areas, but a preference for areas with a high proportion of winter grains and high crop diversity. The trend model confirms these findings with a linear positive effect of diversity on grey partridge population development. Similarly, the pheasant avoids wooded areas but showed some preference for municipalities with open water. The effect of maize was found to be positive at medium densities, but negative at very high proportions. Winter grains, landscape features and high crop diversity are favorable. The positive effect of winter grains and higher crop diversity is also supported by the trend model.

**Conclusions:**

The results show the strong importance of diverse crop cultivation. Most incentives favor the cultivation of specific crops, which results in large areas of monocultures. The results confirm the importance of sustainable agricultural policies.

**Electronic supplementary material:**

The online version of this article (doi:10.1186/s12898-016-0093-9) contains supplementary material, which is available to authorized users.

## Background

Agricultural intensification has led to a dramatic loss in biodiversity from the middle of the twentieth century until now in Europe [1, 2]. Farmland birds suffer especially from these changes. In general, traditional, heterogeneous small structured fields were found to be beneficial for farmland birds across Europe [3]. Agricultural policies, such as EU-directives or national regulations, play a major role in the rapid change of wildlife habitat in agricultural landscapes. A national act in Germany to increase the proportion of biogas implemented in the EEG directive led to a rapid increase in maize cultivation. This act was hold responsible for recent population developments of farmland birds [4, 5]. In 2008, the EU stopped subsidizing set-aside land which had been beneficial to the grey partridge and other farmland birds [6–8]. An increase in production of winter crops, as opposed to more traditional summer grains, was found to be one cause for decreasing segetal flora and to generally less diverse habitats [9, 10].

Most habitat models use abundance data for one to a few years, or point samples over longer periods of time. The use of citizen science data on species densities can provide larger spatial and temporal scales and can be adequate for developing adaptive management programs [11]. A monitoring program is a core component to the management of endangered species; however, it often fails due to financial shortcomings. Many citizen science programs use opportunistic online based databases [12], which often suffer from biased data collection. A long term wildlife survey WTE (Wildtiererfassung Niedersachsen) was established in 1991 with annual questionnaires of hunters in Lower Saxony [13, 14]. With a participation rate of roughly 90 % of hunting districts (equals ca. 8000 districts, 3.7 Mio ha) the hunters built a highly motivated group and provided reasonable estimates of small game species at low cost from a small district to a federal state scale, with confirmed high reliability of estimates of population densities for the grey partridge [15].

The grey partridge (*Perdix perdix*) and the common pheasant (*Phasianus colchicus*) are two small game species typical of the agricultural landscapes in Central Europe. The grey partridge is listed as “endangered” in the Red List of threatened birds in Lower Saxony [16]. As a typical species of traditional small structured farmland the grey partridge was originally widespread in Lower Saxony, but less so in the densely wooded sub-mountainous areas of southern Lower Saxony and the sandy heathlands of eastern Lower Saxony. Today, we find great differences between areas of intensive agricultural use with 0.3:0.9 pairs/km^2^ and <0.2 pairs/km^2^ near the North Sea and in the South. In 2014 the grey partridge still occurred in 41 % of hunting districts [17]. The overall population size in Lower Saxony was estimated at 25,000 breeding pairs in 2008 [18].

The pheasant is not listed on the red list. It was introduced to Central Europe by the Romans and is now a typical species of arable land, pastures and reed edges of open waters. By limiting hunting to pheasant cocks, the actual sex ratio is at about 1:2 for cocks and hens [17]. In the main distribution areas the hen density is between 8:12 hens/km^2^. In the wooded areas of southern and eastern Lower Saxony the density is at <5 hens/km^2^.

Although reintroductions and hunting may confound population trends; for Lower Saxony they currently play a minor role. The grey partridge, specifically, is only released in very limited instances. Pheasant releases have also decreased considerably. As assessed in 2008, pheasants are released in only 3 % of hunting districts amounting to roughly 5000 per year in the entire area of Lower Saxony [17]. Shooting time for the pheasant is between 1 Oct–15 Jan and 16 Sep–30 Nov for the grey partridge. Since 2012 the Hunting Association of Lower Saxony has encouraged a complete voluntary stop of grey partridge hunting in response to continuous population decline. Hunters have followed this recommendation with very few exceptions [17]. Pheasant hunting still occurs in two thirds of hunting districts; however the hen density should be unaffected. Whereas many studies use hunting bags to model trends [i.e. 19–21], their reliability for small game species is questionable. Differences in motivation biotic and abiotic, as well as legislative changes may influence hunting bags more than changes in population densities [14, 17].

Due to agricultural intensification and two extreme winters, grey partridge and common pheasant populations have experienced dramatic declines since the late 1970s. The common pheasant recovered partly due to artificial reintroductions but the grey partridge has continued to and is currently declining. Since 2006 for the grey partridge and since 2008 for the pheasant, parts of Lower Saxony have seen a rapid decline in population densities. Although their population trend is not identical, the two species show some parallelism in habitat preferences and a common cause of decline is suspected. In analyzing both trends separately we hope to validate the reliability of results.

**Fig. 1:**
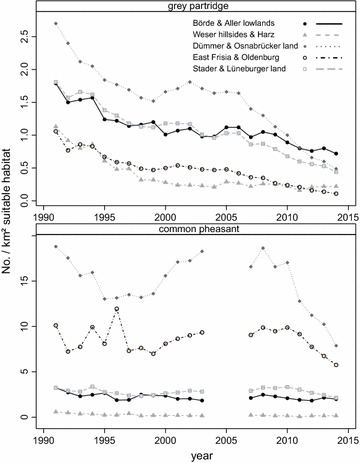
Mean number of grey partridge breeding pairs and common pheasant hens per km^2^ open land per municipality. As part of the wildlife survey (WTE) estimates are recorded through annual questionnaires of local hunters summarized for five natural regions (following [68], modified by E. Strauß) with different dominant landscape features from 1991:2014. For the pheasant there is a gap of 3 years between 2004 and 2006

The areas traditionally inhabiting the highest abundances, most notably the Dümmer and Osnabrücker land in western Lower Saxony, are also the areas with the steepest decline (see also Fig. 1). Resource limitation increases competition, and with scarcer resources, density increases the severity of competition. For the grey partridge, a density dependence of reproductive success was found across Europe [22–24]. After hatching, grey partridge and pheasant chicks rely on insects for survival (first 2–6 weeks for the grey partridge and 2–7 weeks for the pheasant), and beetle banks were found to be beneficial in England [25]. The historic decline of farmland birds, including grey partridges, was due to a decrease in insect diversity and abundance caused by pesticides ([26, 27] and publications therein). Thus, a likely cause may be specific agricultural practices i.e. pesticides that reduce insect and consequently bird abundance [28, 29]. Spatially and temporally explicit data on pesticide application are difficult to obtain. However, with specific crops being particularly unfavorable this may point to adverse cultivation practices. The latest decline causes may be different from the well-established causes as seen above; one example being the preference for maize cultivation for use as biofuel which was politically provoked. Over the last 8 and 10 years, trends at municipal scale were compared to try and establish causes by comparing changes in crop proportions per municipality.

In 2005, Lower Saxony implemented a directive of the European Union to establish a program to monitor and control spatially explicit data on field crops and agricultural subsidies. For research projects depersonalized data of crops can be accessed for research. These are the most detailed data on agricultural use in the European Union and give detailed information on roughly 90 % of agriculturally used lands in Lower Saxony (LEA-Portal (see below) as measure of 100 % area).

In this paper we modelled both the habitat preferences and spatially explicit trends and its causes for the grey partridge and the common pheasant. The first aim is to explain the overall density with landscape traits and functional crop groups and discuss differences in habitat preferences. The second approach is aimed at explaining the negative trends observed over the last 8 and 10 years. Specifically, we expect negative effects of (1) increasing maize cultivation (2) decreasing proportions of set aside fields (3) increasing proportions of winter crops, (4) the general intensification as indicated by declining crop diversity and increasing field sizes.

## Methods

The federal state of Lower Saxony (Germany) has a total area of 47,620 km^2^, of which 60.2 % are in agricultural use and 21.6 % is forested land. 2.3 % of the area is open water. The remaining area is dominated by industrial, traffic and housing areas. North-west Lower Saxony adjoins the North Sea, in the south-east the Harz Mountains rise up to 1000 m elevation. Main habitat conditions differ considerably between natural regions. The north east has predominantly sandy soils and is generally dominated by coniferous forests (Additional file 1: Figure S1a) and arable lands with high proportions of potatoes. East Frisia at the North Sea has the highest proportion of open water (Additional file 1: Figure S1b). The west is mostly dominated by animal husbandry and cattle farms, especially in the north where there are high proportions of grassland (Additional file 1: Figure S1c). In the west there are industrial indoor poultry and pig farms and the crops have the highest level of diversity as indicated by the Shannon index (see below, Additional file 1: Figure S1d). The arable land of the southeast Börde and Aller lowlands has predominantly fertile soils (Chernozems) with a dominating cultivation of wheat and sugar beet. Here the land is poorly structured with large field blocks and few hedges and tree rows (Additional file 1: Figure S1e, f). The climate is oceanic in the west but increasingly continental towards the east. The lowest mean annual temperatures of 6 °C, and at the same time the wettest climate with >1000 mm annual precipitation sums, are recorded in the Harz Mountains. The warmest places are in the westernmost areas at >8.5 °C and the driest area is in the east with <600 mm annual precipitation.

Lower Saxony is politically structured in 47 administrative districts and 455 greater municipalities which are the sample units in the analyses (see below).

### Data

Wildlife survey: Lower Saxony is divided into approximately 9000 hunting districts. Since 1991 holders of the hunting districts have provided estimates of wildlife in their hunting ground for a number of species including the grey partridge and common pheasant. Participation of hunting district holders was high throughout the years 1991–2014 and ranged between 80 and 90 % of hunting districts (6151–8300). Also, over 90 % of the huntable area of Lower Saxony was recorded (43,000 km^2^). As rigorous quality control for the grey partridge, the estimates were evaluated and directly compared to the counts of ornithologists and found to be reliable [15]. Unfortunately a comparable evaluation of pheasants was not undertaken; however even if the accurateness of estimates in absolute numbers cannot be guaranteed, the trend over the years is likely to portray the real population trend.

The counts from all hunting districts were aggregated to 420 greater municipalities (excluding 35 municipalities that were either unincorporated land as well as the islands in the North Sea). The mean huntable area of hunting districts was 500 ha (min 75 ha, max 4877 ha in 2012). The total area between years stayed almost constant as a high participation was achieved in every year. Hunting district holders report huntable area, area of wooded land and open land. For the pheasant district holders reported numbers of pheasant hens and cocks in spring for their hunting district, for the grey partridge they estimated breeding pairs. Pheasants and grey partridge densities were extrapolated to numbers/pairs/km^2^ open land for the grey partridge excluding water bodies. In the years 2003:2005 the pheasant population was not assessed. In 2006 participation for the pheasant was lower and thus the year was also omitted from analyses. Tests for plausibility were run every year and obvious mistakes were removed.

IACS: In 2005 the Integrated Administration and Control System (IACS) of the European Union was introduced in Lower Saxony. Since then data on all arable fields, or of all farmers that receive subsidies in any form, have been documented. For Lower Saxony, that comprised roughly 90 % of all agricultural land as indicated by the total area of the LEA-Portal (http://sla.niedersachsen.de/landentwicklung/LEA/). IACS data were provided by the SLA (“Servicezentrum Landentwicklung und Agrarförderung” in Lower Saxony). For each field in the database we obtained data on crop type, the size of the field and the municipality it was situated in. Over 10 years this roughly amounts to 9.5 million fields. For reasons of privacy protection no further details on geographic locations or field ID were provided. Thus, no data on crop rotation or neighborhood statistics were possible. Data were summed to percentage area agricultural land per municipality. IACS data are grouped in over 164 different crops and had to be summarized into ecologically sensible groups (Additional file 2: Table S1 for details). From those groups the Shannon index (see below) was calculated. Further simplification of crop groups was used for the habitat and trend models (see below).

The Shannon Index is a standard measure for alpha diversity in vegetation science [30]. Here we use the same metric for crops within municipalities instead of species abundance per vegetation unit. Thus it is defined as:

$$H_{s} = - \mathop \sum \limits_{i = 1}^{S} p_{i} * \ln p_{i}$$

Hs = diversity of s municipalitiess, s = no. of crops, p_i_ = relative abundance of the i-th crop from 0, 0 to 1, 0. The Shannon Index was calculated in the R package vegan [31].

Mean field size was calculated per municipality. Field block size and landscape features were achieved from the LEA-Portal (http://www.sla.niedersachsen.de/landentwicklung/LEA/) for 2014 only; therefore they were only used for the habitat model. Percent land cover of main landscape features like woodland, water expanse and grassland were obtained from the LSA (Landesamt für Statistik Niedersachsen) (http://www1.nls.niedersachsen.de/statistik) these were available for the years 2005, and 2009–2014. For the missing years 2006–2008 the values for 2005 were replicated to decrease unduly reduction in sample size of the overall habitat models. This seemed like a moderate flaw as these broad categories only changed marginally over the 10 years. E.g. woodland cover changed in 85 % of municipalities to less than 1 % of total area, the maximum value was less than 6 % change of total municipal area.

Before starting statistical modeling extensive data mining was applied and all data were examined for plausibility and confounding effects. Multivariate analyses (i.e. PCA, CCA, NMDS and indicator species analyses) were undertaken to find specific crops that might be used to explain trends in grey partridge or common pheasant densities. Summer barley and triticale explained the highest proportion in the multivariate tests, which could not be supported in univariate tests. Therefore, we decided to analyze functional groups of crops instead of individual crops, as a number of explanatory variables had to be reduced to enable conversion of models.

### Statistical analyses

All data preparation and analyses were conducted in R 3.1.2 [32]. The statistical models were conducted in the R package mgcv [33, 34]. Model selection on fixed effects was accomplished by AIC comparisons using maximum likelihood estimations (see Additional files 2, 3: Tables S2–S5 for an overview on all tested candidate models and the process of model selection).

### Habitat modeling

The relations between response and explanatory variables were partly non-linear, thus additive mixed models were applied to model density data. Municipality was integrated as random effect. An autoregressive correlation structure was found to improve the model fit as measured by AIC comparisons. Latitude and longitude as two-dimensional tensor product smoothers were incorporated to account for spatial autocorrelation.

For the fixed effects, the following parameters were included: year as factor, winter grain, summer grain, maize, set aside, woodland (including deciduous woodland and coniferous forest), water expanse (including all water courses, rivers, canals, lakes, the shore of the North Sea and swamps). Grasslands are highly negatively collinear with the Shannon Index (r = −0.7). Since crop diversity resulted in better model fit than grassland we tested the Shannon Index rather and followed the |r| > 0.7 rule [35]. The full model had the structure (Eq. 1):

1 $$ \begin{aligned}&E\left[ {\frac{{No.\,pheasant\,hens} -{{No.\,grey\,partridge\,breeding\,pairs}}}{{ha\,potential\,habitat}}} \right]  \\ \quad &= f\left( {{winter\,grains}\, \%_{i} } \right) + f\left( {{summer\,grains} \, \%_{i} } \right) + f\left( {{maize}\, \%_{i} } \right) + f\left( {{mean\,field\,block\,size}_{i} } \right) \\ \quad &+ f( {{landscape\,features}\, (\%) }/(field\, block \,area)_i)+f(set\, aside\, fields \%_{i}) +   \\ \quad &+ f\left( {{Shannon\,index}_{i} } \right) + f\left( {{woodland}_{i} } \right) + f\left( {{water\,courses}_{i} } \right) + {factor\;(year)} \\ \quad &+ \,{random}\left( {{municipality}} \right) + f\left( {{latitude}_{i} * {longitude}_{i} } \right) + \varPhi e_{i - 1} + \in_{i}.\end{aligned} $$

The response variables are: 1. Grey partridge breeding pair/km^2^ open land (excluding water bodies). 2. Pheasant hen numbers/km^2^ open land. The term *f()* indicates a smooth term (Spline-Regression), *random()* a random effect structure. The term $\varPhi e_{i - 1} + \in_{i}$ describes an autoregressive term to control for temporal autocorrelation.

Due to some missing values in the response or explanatory variables, the total sample size of the model resulted in 3877 observations for the grey partridge model and 3101 observations in the pheasant model.

### Trend analysis

In order to model the trend we calculated spearman rho correlations between all tested parameters and year for each municipality separately. The rho values of grey partridge and common pheasant were used as dependent variables and rho of all agricultural crops as explanatory variables. For the grey partridge the correlation was calculated of 10 values (10 years) for the pheasant only 8 years were available (see above). We favored the rho coefficient over Pearson r or the estimates of (generalized) linear models. These are more sensitive and require stricter assumptions in model fit. A correlation coefficient calculated from 8 or 10 values is a rather crude value and rho is heavily influenced by the 1st years of survey. A dramatic decline starting in the middle of the observation period would result in a weak rho coefficient. However, we argue that a decline only over a few years is not necessarily a real trend and an underestimation is potentially ecologically worthwhile. Rho as dependent variable was arc sine transformed to account for upper and lower boundedness. A GAM (generalized additive model) was fitted to account for non-linear trends in the data.

Full model in Eq. 2:

2 $$\begin{aligned}&{arc\,sine}\left[ {{rho\,pheasant\,hens} - {rho\,grey\,partridge\,breeding\,pairs}} \right] \\ \quad &= f\left( {\textit{rho winter grain}_{i} } \right) + f\left( {\textit{rho summer grain}_{i} } \right) + f\left( {\textit{rho maize}_{i} } \right) \\ \quad & +f(\textit{rho mean field size}_{i} ) + f\left( {\textit{rho Shannon Index}_{i} } \right) \\ \quad & + f(\textit{rho set aside fields}_{i} ) + f\left( {{latitude}_{i} * {longitude}_{i} } \right) \end{aligned}$$

The response variables are: 1. Rho grey partridge breeding pair/km^2^ open land (excluding water bodies) calculated over 10 years. 2. Rho pheasant hen numbers/km^2^ open land calculated over 8 years. The term *f()* indicates a smooth term (Spline-Regression).

Main landscape types were not tested in the trend model as changes were minor and not all years were available (see above). Also data for landscape features and field block size were only available for 2014, thus, we could not quantify changes. Latitude and longitude as two-dimensional tensor product smoothers were incorporated to account for spatial autocorrelation.

The total sample size, due to missing values resulted in 413 observations in the grey partridge model and 395 observations in the pheasant model.

## Results

Both species showed a dramatic decline over the years 1991–2014 (Fig. 1). The population collapse for the grey partridge was most severe, and until 2014 its population density decreased about 60–90 % as compared to 1991 (Fig. 1a). The decline differed between geographic regions and showed that the pheasant had larger fluctuations with a phase of increasing population densities between 1995 and 2005 (Fig. 1b). But ultimately, the pheasant lost between 36 and 90 % of its original population size between 1991 and 2014.

**Fig. 2:**
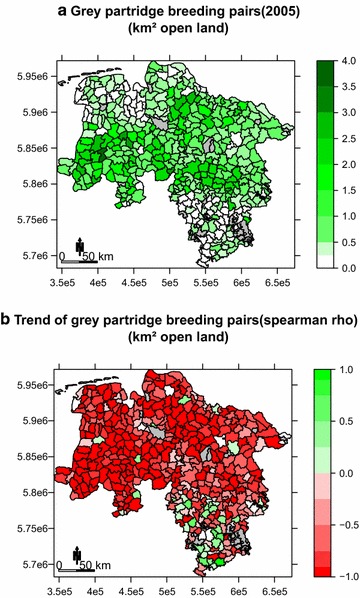
**a** Grey partridge breeding pair density 2005; **b** grey partridge breeding pair spearman rho correlation coefficients (2005–2014) per municipality in Lower Saxony. *Red* indicating negative population trends green positive trends. *Grey* no data (Cartographic base: GeoBasis-DE/BKG 2002, data source: wildlife survey)

**Fig. 3:**
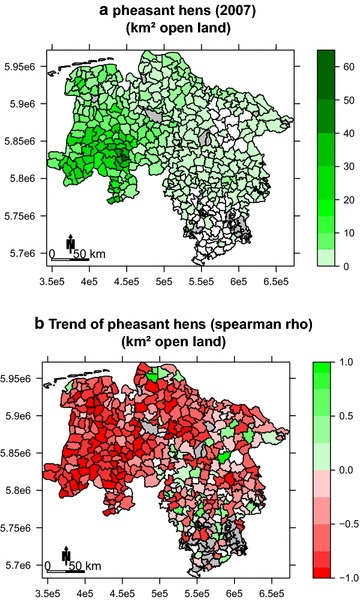
**a** Common pheasant hen density 2007; **b** common pheasant hen spearman rho correlation coefficients (2007–2014) per municipality in Lower Saxony. *Red* indicating negative population trends green positive trends. *Grey* no data (Time span differs to the grey partridge. Pheasants were not recorded in 2005. In 2006 data were sparse and thus also omitted from analyses) (Cartographic base: GeoBasis-DE/BKG 2002, data source: wildlife survey)

Between 1991 and 2005 grey partridge density was highest in western and central Lower Saxony (Figs. 1a, 2a). Also, for the shorter study period (2005–2014) that was used for the habitat and trend models, the grey partridge showed a strong negative trend over the entire study area (median: rho = −0.79, 1st and 3rd quantiles: rho = −0.94 and −0.47), with the severest decline in western Lower Saxony (Fig. 2b). Since the start of data collection in 1991 the common pheasant had its highest population densities within the westernmost parts of Lower Saxony (Figs. 1, 3a), it also showed a negative trend between 2007 and 2014; however, it was somewhat less severe (median: rho = −0.60, 1st and 3rd quantiles: rho = −0.81 and −0.17). The pheasant also declined most severely in the westernmost areas of Lower Saxony (Fig. 3b).

### Habitat modeling

**Fig. 4:**
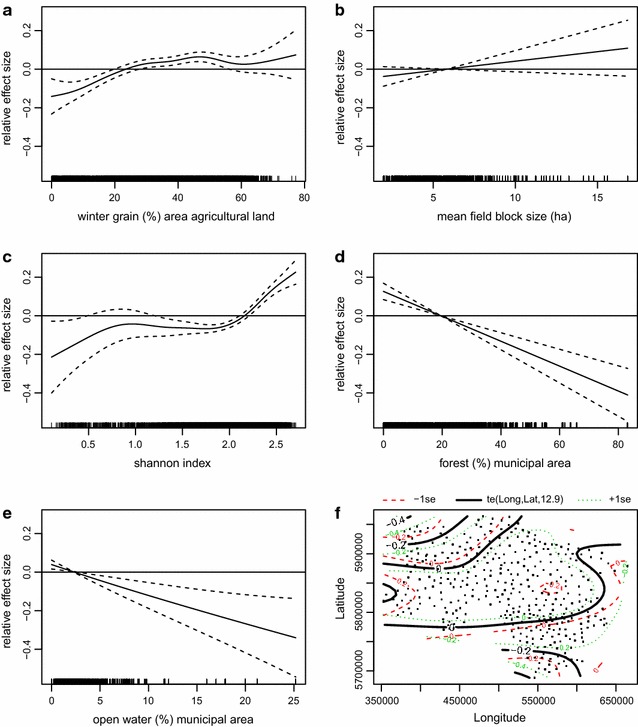
Minimum adequate habitat model of grey partridge breeding pairs. Figure displays results of GAMM showing significant smoothers: **a** winter grain (%) agricultural land, **b** mean field block size, **c** Shannon Index, **d** % forest/municipal area, **e** % open water/municipal area, **f** longitude × latitude. R^2^ adjusted = 0.48

The minimum adequate model for the habitat model of grey partridge breeding pairs underlines the dramatic loss in grey partridge abundance, with all years, except for 2006, having significantly lower densities than 2005 (Table 1, p < 0.001). When percentages of winter grain are <20 %, grey partridges are less abundant. In municipalities with higher proportions the model shows overall positive responses, however when it is above 55 %, the effect is non-significant and the standard error gets larger (Fig. 4a). The non-significant smoother for field block size should not be overestimated; nonetheless it improves model fit and vaguely points to a preference of relatively large field blocks of >6 ha (Fig. 4b). As the second most important smoother (Table 1; F = 20.7, p < 0.001) for crop diversity per municipality, the Shannon index shows that highly diverse municipalities are of benefit for the grey partridge (Fig. 4c). The grey partridge is rare in areas with a high proportion of forest/woodland, which is at the same time the most important coefficient (Table 1; F = 35.6, p < 0.001), and also a negative response to water expanse (Fig. 4 d, e). The tensor product of longitude by latitude shows the high density areas in central Lower Saxony and the lower abundances in the north and the south (Fig. 4f). The model with R^2^ adjusted at 0.48 explains roughly half the variance.

**Table 1:** Summary of GAMM showing habitat preferences of grey partridge breeding pairs as modelled by  % share of arable crop groups and other important landscape features per municipality

	Estimate	SE	t value	Pr (>|t|)	
*Parametric coefficients*					
(Intercept)	0.617	0.012	51.546	<0.001	***
Year = 2006	−0.010	0.009	−1.198	0.231	
Year = 2007	−0.073	0.009	−7.969	<0.001	***
Year = 2008	−0.057	0.009	−6.228	<0.001	***
Year = 2009	−0.103	0.009	−11.160	<0.001	***
Year = 2010	−0.130	0.009	−13.776	<0.001	***
Year = 2011	−0.175	0.010	−17.988	<0.001	***
Year = 2012	−0.208	0.010	−20.047	<0.001	***
Year = 2013	−0.207	0.009	−21.897	<0.001	***
Year = 2014	−0.245	0.010	−25.325	<0.001	***

	edf	Ref.df	F	p value	
*Approximate significance of smooth terms *					
s(Winter grain)	5.704	5.704	5.586	<0.001	***
s(Field block size)	1.000	1.000	2.245	0.134	
s(SHANNON index)	5.900	5.900	20.662	<0.001	***
s(Forest)	1.000	1.000	35.606	<0.001	***
s(Water expanse)	1.164	1.164	11.704	<0.001	***
te(Longitude, latitude)	12.905	12.905	8.844	<0.001	***

R^2^ adjusted = 0.48

**Fig. 5:**
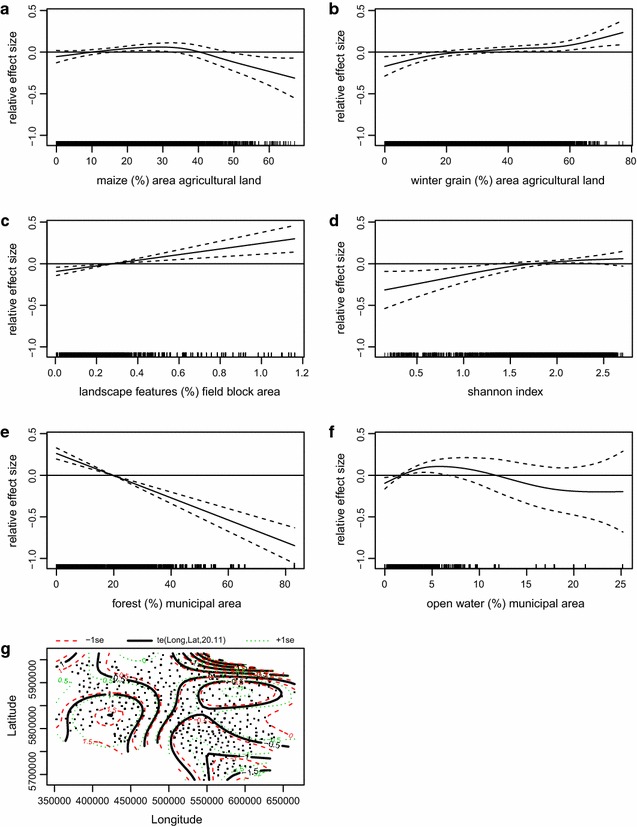
Minimum adequate habitat model of the common pheasant hens. Figure displays results of GAMM showing significant smoothers: **a** % maize/agricultural area, **b** % winter grains /agricultural area, **c** % landscape features/field block area, **d** Shannon Index, **e** % forest/municipal area, **f** % open water/municipal area, **g** longitude × latitude. R^2^ adjusted = 0.82

The minimum adequate GAMM for pheasant hens shows a unimodal relationship to percentage of maize per area. Between approximately 15 and 35 % the effect is moderately positive, whereas at the highest percentages, maize has a negative effect on pheasant hen densities (Fig. 5a). In contrast, the effect of winter grains is mostly positive. Below 20 % the effect is negative; above 40 % it is positive (Fig. 5b). The effect of landscape features shows a linear positive trend (Table 2; edf = 1, p < 0.001, Fig. 5c). Municipalities with a low Shannon index as measure for crop diversity host fewer pheasants than more diverse areas. The positive effect of highly diverse municipalities is not very pronounced, however, the negative effect of municipalities with few crop types is more evident (Fig. 5d). Municipalities with a high proportion of woodland or forests are generally unfavorable habitats for pheasants (Fig. 5e) and at the same time the most important smoother to describe pheasant hen abundance (Table 2; F = 61.8, p < 0.001), whereas the percentage of some water (approximately 1–7 %) is positive in general. At values higher than 8 percent the sample size is very low and thus also the standard error is large (Fig. 5f). Longitude and latitude generally show a west east gradient with highest density in the westernmost areas, with lower densities near the coast and lowest densities in the north and south east of Lower Saxony (Fig. 5g). R^2^ adjusted is with a value of 0.87 comparably high.

**Table 2:** Summary of GAMM showing habitat preferences of common pheasant hens as modelled by  % share of arable crop groups and other important landscape features per municipality

	Estimate	SE	t value	Pr(>|t|)	
*Parametric coefficients*					
(Intercept)	1.38362	0.01975	70.069	<0.001	***
Year = 2008	0.06254	0.01075	5.82	<0.001	***
Year = 2009	0.02158	0.01388	1.555	0.120	
Year = 2010	0.03784	0.01668	2.269	0.023	*
Year = 2011	−0.04684	0.01871	−2.504	0.012	*
Year = 2012	−0.092	0.01968	−4.676	<0.001	***
Year = 2013	−0.15274	0.01955	−7.815	<0.001	***
Year = 2014	−0.23899	0.02024	−11.81	<0.001	***

	edf	Ref.df	F	p value	
*Approximate significance of smooth terms*					
s(Maize)	4.331	4.331	6.007	<0.001	***
s(Winter grain)	4.126	4.126	3.870	0.005	**
s(Lanscape features)	1.000	1.000	14.074	<0.001	***
s(Shannon index)	2.391	2.391	3.994	<0.013	*
s(Forest)	1.000	1.000	61.792	<0.001	***
s(Open water)	3.304	3.304	4.347	0.004	**
te(Longitude, latitude)	20.112	20.112	57.676	<0.001	***

R^2^ adjusted = 0.87

### Trend analysis

**Fig. 6:**
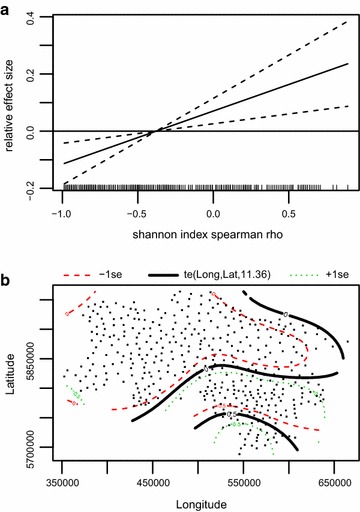
Minimum adequate model of grey partridge breeding pair trends in Lower Saxony. Figure displays results of GAM showing significant smoothers. **a** Trend of Shannon Index, **b** longitude × latitude. Explained deviance = 32.1 %

Modeling the rho coefficient of grey partridge trend resulted in a model underlining the importance of diverse agricultural crops (Fig. 6a). With an F value of 10 its importance is even higher than the spatial effect (F = 8, Table 3). Here again the spatial trend indicates the highest population losses in the westernmost areas and more stable conditions in southern Lower Saxony (Fig. 6b). Explained deviance of the grey partridge trend model is at 31.2 %.

**Table 3:** Summary of grey partridge trend model showing parametric coefficients and summary of smooth terms

	Estimate	SE	t value	Pr(> |t|)	
*Parametric coefficients*					
(Intercept)	−0.755	0.024	−30.980	<0.001	***

	edf	Ref.df	F	p value	
*Approximate significance of smooth terms*					
s(rho Shannon index)	1.000	1.000	10.036	0.002	**
te(longitude, latitude)	11.370	14.100	8.053	<0.001	***

Explained deviance 32.1 %

**Fig. 7:**
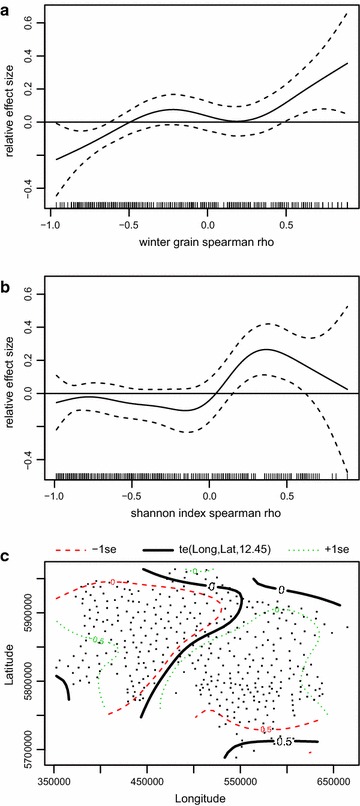
Minimum adequate model of common pheasant hen trends in Lower Saxony. Figure displays results of GAM showing significant smoothers. **a** Trend of winter grains, **b** trend of shannon index, **c** Longitude × Latitude. Explained deviance = 36.2 %

The minimum adequate GAM for common pheasant trend shows an overall positive effect of increasing winter grain proportions. In municipalities with a steady decrease in winter grains the population trend of the common pheasant is negative, whereas in municipalities with generally increasing proportions of winter grain the population trend is positive (Fig. 7a). Similarly in municipalities with increasing crop diversity, pheasant hen abundance generally increases. However, the results also show that municipalities with rho of >0.6 are rare, thus confidence intervals get larger and the trend is not significant at the highest values (Fig. 7b). The two dimensional smoother of longitude and latitude indicate the highest decrease in the westernmost areas of Lower Saxony and a slight increase for the southernmost areas (Fig. 7c). The spatial trend explains most of the variance of the model (F = 6.2), followed by winter grain F = 3.5 and Shannon index F = 2.4. The model generally explains 36.2 % of deviance (Table 4).

**Table 4:** Summary of trend model for common pheasant hens showing parametric coefficients and summary of smooth terms

	Estimate	Std. Error	t value	Pr(> |t|)	
*Parametric coefficients*					
(Intercept)	−0.500	0.024	−20.960	<0.001	***

	edf	Ref.df	F	p value	
*Approximate significance of smooth terms*					
s(rho winter grains)	3.665	4.550	3.460	0.006	**
s(rho Shannon Index)	5.220	6.353	2.366	0.030	*
s(longitude, latitude)	12.448	15.134	6.174	<0.001	***

Explained deviance 36.2 %

## Discussion

### Habitat modeling

Generally, the data were found to be well suited to model common pheasant and grey partridge density. With a value of 0.87 of R^2^ adjusted, the pheasant habitat model explains most of the variance. The grey partridge model explains considerably less, but still, a value of R^2^ adjusted = 0.48. However, the coordinates were the second most important explanatory variables for the pheasant model and are among the more important variables for the grey partridge model but do not explain habitat preferences. They were necessary to control for spatial autocorrelation and may indicate that some important explanatory variables are missing from the model [36, 37].

The percentage of woodland per municipal area was the most important explanatory variable for both species. As both are typical species of open landscapes, [38] a negative effect of higher proportions of woodland is straightforward. The remaining parameters of landscape features, field block size, Shannon index, water expanse, winter grain and maize are all of similar importance and are the more conclusive results. In many respects, the grey partridge’s habitat preferences are comparable to the pheasant. They also avoid municipalities with a higher percentage of woodland. However they prefer municipalities with <3 % open water bodies. Pheasants show some preference to municipalities with higher proportions of open water, which is a plausible result. If available pheasants are often seen in reed (*Phragmites australis*) edges of water courses and lakes [38]. These findings are well-known differences in habitat selection [39].

Winter grains were beneficial to both species and may provide shelter and food over the winter which may increase winter survival. Grey partridges need high energy food in winter, especially if winters are harsh [40]. However, attempts with food supplementation over the winter did not show any improvement of reproductive success in the following reproductive season in northern France, [41] and autumn and winter diet analyses support no fodder scarceness for grey partridges during these seasons in Poland [42].

Maize was found to be favorable for the pheasant up to a percentage of roughly 20–30 % of arable land. For higher percentages (>50 %) it has negative effects, however the sample size was somewhat low at the highest percentages and the standard error increased. This is not as convincing of an effect as previously found for the farmland birds skylark (*Alauda arvensis*), yellow wagtail (*Motacilla flava*), corn bunting (*Miliaria calandra*) and northern lapwing (*Vanellus vanellus*) [4]. Overall, maize offers landscape and functional diversity in contrast to areas only dominated by winter grains or grassland. Landscape diversity was found more important than low intense farming techniques for vertebrates including farmland birds [43]. Functional diversity in habitat types also increases functional diversity of insects [44] and increases overall densities and biomass of insects [45]. The grey partridge, however, does not show any preference or avoidance of maize. At different spatial or temporal scales, the effects might be different and we do not rule out adverse effects of increasing maize cultivation. Municipalities with high diversity of crops were of advantage for both species.

Landscape features such as hedges and tree rows provide shelter for the pheasants and a positive effect is plausible [46]. The overall picture of the landscape feature data seems adequate in that the areas of Lower Saxony that have higher proportions of hedges also apply for more subsidies, but it is difficult to evaluate whether or not it is proportionate to the actually present features. The grey partridge does not show any relation to landscape features in the models. Other authors describe a relation of hedgerows or permanent cover and grey partridges [47, 48]. These differences may be due to scale, differences in structures between England, Poland and Germany or the known deficits of the used landscape feature data. The grey partridge, however, shows preferences to relatively large field blocks. This may be an effect of adaptation to typical arable land with generally larger fields. Historically and geographically, it was precluded that smaller fields are of advantage for farmland birds [3]. However, northern France supported our findings with on average larger fields encompassing higher population densities of the grey partridge [49]. Whether the grey partridge is an exception, or the results are confounded with underlying effects is difficult to decide and may be of limited importance, considering the non-significant effect. Yet, it may be potentially interpreted as beneficial as grey partridges use the middle of fields during the night as a predator avoiding behavior [50]. Thus, with larger field blocks they may escape predators more easily as most nocturnal predators predominantly search for prey at field margins.

### Trend analysis

Increasing winter grains were found to have a positive effect on pheasant population growth, but no effect on grey partridge trends. Areas with the most fertile soils dominated by winter grains i.e. most notably the Börde and the southernmost areas supporting the lowest population densities (Fig. 2a), showed overall inconsistent trends but no convincing population decline (Fig. 2b).

The Shannon index is the most important smoother in the grey partridge model and also significant in the pheasant model. But the two dimensional smoothers explain most of the variance in the pheasant model and improves model fit in the grey partridge model.

The CAP (Common Agricultural Policy) reform after 2013, which installed mandatory greening measures, should increase crop diversity and thus alleviate negative population trends. At this stage it is too early to confirm any benefits and unfortunately most experts expect small effects on sustainability and biodiversity gain [51, 52], therefore we encourage a more combined effort of rural actors to increase a diversity of ecological niches in agricultural lands.

## Conclusion

The most conclusive result is the overall importance of diverse crops, which supports our initial expectation and the findings of several studies [e.g. 3, 53]. Other indications of a general intensification of agricultural land use did not show as strong effects. Mean field size increased marginally (from 0.39 to 0.4 ha, including non-typical crops) over the 10 years, but did not explain grey partridge or pheasant habitat or trend models. Data on landscape features are not complete; nevertheless pheasants show some preference to areas with a higher abundance of hedges and tree rows, but we have no data on changes of feature abundance.

Generally within the last 10 years, most signs of agricultural intensification including crop yield, proportion of cultivated to uncultivated land, pesticide and nitrogen application and livestock density have stabilized in most of western Europe or even decreased [54], however this may not rule out higher effectivity of applied compounds. Both, insecticides as well as herbicides have adverse effects on farmland birds impeding the manifold direct and indirect benefits of weeds and invertebrates to wildlife [45]. Food availability models [55] improve habitat models and fodder scarceness for chicks [56] or nest site limitation [57] are possible density dependent causes that can be summarized under the term of resource limitation. Fodder limitation in autumn and winter was found to be unproblematic for grey partridges in Poland [42]. But a density dependent reproductive success was found for the grey partridge at larger population densities in northern France, which was attributed to a lack of favorable habitats [22]. Chick survival rate was found to be crucial for the population decline in several European countries [24, 58, 59] and decreased significantly after the introduction of pesticides [60]. Pheasants at very high abundances may also alter insect abundance [61], however comparable densities are not found in the study area.

Specific crops, especially maize, are held responsible for the latest decline of small game species in Germany. The pheasant habitat model found some weak indication of negative impacts of very high dominance of maize, but the trend model did not confirm a negative effect of increasing maize cultivation. Maize cultivation had already increased before 2005, consequently some effects may have escaped our observation and population declines may have a delayed response to the actual change. Moreover, it is likely that a combination of adverse effects may lead to a dramatic decline rather than a single cause [62]. However, we believe that the widely accepted detrimental effect does not apply to these two species. At different spatial scales unfavorable attributes of maize may be more evident though, as scale significantly affects pheasant habitat models [63]. Radio tracked grey partridges use maize fields, but prefer wild flower strips and sunflower fields in summer and hedges in spring and winter [64]. Despite the lack of significant effects of maize, the analyses showed that monocultures are negative for population trends and municipalities with a 50–80 % of agricultural area with maize cultivation are certainly undesirable for wildlife as shown by the significant effects of crop diversity.

Set-aside fields did not show any significant relation to grey partridge or pheasant trends. Since 2008, no subsidies were given to most categories of set-aside fields, thus most categories of set-aside fields no longer turn up in the statistics. Most farmers converted their fields to arable land; however, some might have left it as it was; which we cannot assess correctly. As an overall consequence, our data might overestimate the negative trend and variations between municipalities may not show up adequately.

Winter grains were found to be beneficial, whereas summer grains showed no effect. Summer grains are relatively strongly correlated to Shannon index (r = 0.4 for the habitat variables and r = 0.56 for the rho values). Thus, it may be difficult in parts to differentiate between the higher diversity and the effect of summer grains. Generally cereals provide nutrient rich fodder, which applies to both groups.

Other causes of decline were not tested. Predators were discussed as crucial for population dynamics [65]. The fox however, as main predator of pheasants in Lower Saxony (Voigt unpublished data) were observed to be relatively stable over the time period [17]. A potential effect of climate change should not be locally concentrated, but may explain a general regression of species distribution ranges [66, 67]. For the population dynamic of the two species, climate change was relatively improbable as an effect as Lower Saxony is not at the edge of their climatic niche.

A density dependent decline may also be due to an epidemic. Municipalities with the strongest negative trend are also the municipalities with the highest density in poultry farms within Germany. Only the administrative district of Vechta inhabits over 4 million laying hens in an area of 2018 km^2^. Mutual infections between wild galliformes and laying hens are here one among many possibilities. In pheasants ongoing investigations found a high amount of antibodies against infections also typical of poultry farms. Whether these are attributable to the same strains and whether they are at all pathogenic to pheasants is currently being investigated. Chicks were especially found to suffer from diverse infections and parasites [17].

## Additional files

10.1186/s12898-016-0093-9 Mean proportions of main habitat types (% forest, % open water, % pastures and meadows, shannon index, field block size, % landscape elements) per natural region (Meynen et al. 1962, modified after Strauss).
10.1186/s12898-016-0093-9 List of crops eligible to payments schemes between 2005 and 2014.
10.1186/s12898-016-0093-9 Process of model selection for the Habitat (GAMMs) and Trend (GAMs).

## Abbreviations

IACS: Integrated Administration and Control System
WTE: Wildlife survey Lower Saxony (Wildtiererfassung Niedersachsen)
EEG: renewable energy directive (Erneuerbare-Energien-Gesetz)
LEA-Portal: internet portal for rural development and agricultural subsidies (Landentwicklung und Agrarförderung)
SLA: service center for rural development and agricultural subsidies (Servicezentrum Landentwicklung und Agrarförderung)
PCA: principal component analysis
CCA: canonical correlation analysis
NMDS: non-metric multidimensional scaling
GAMM: generalized additive mixed model
GAM: generalized additive model
CAP: common agricultural policy
