# Supplementary material for: Crop diversity loss as primary cause of grey partridge and common pheasant decline in Lower Saxony, Germany
Source: BMC Ecol. 2016 Sep 9;16(1):39. doi: 10.1186/s12898-016-0093-9 (PMC5016946; doi:10.1186/s12898-016-0093-9)
Supplement: Supplementary file 1 — 10.1186/s12898-016-0093-9 Mean proportions of main habitat types (% forest, % open water, % pastures and meadows, shannon index, field block size, % landscape elements) per natural region (Meynen et al. 1962, modified after Strauss). [file 12898_2016_93_MOESM1_ESM.pdf]

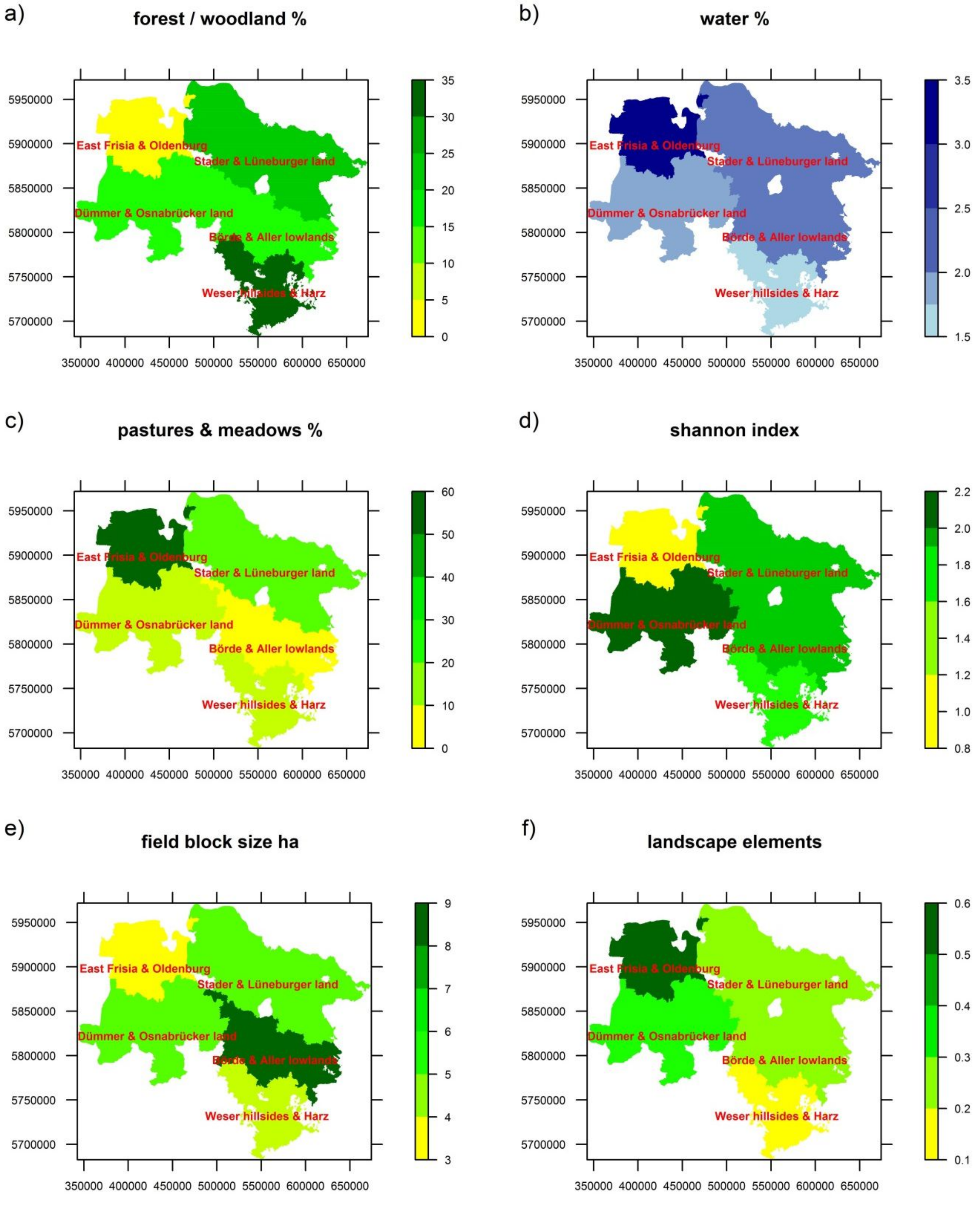

Figure S1: Mean proportions of main habitat types per natural region (Meynen et al. 1962, modified after Strauss). The five regions comprise: North-West: East Frisia and Oldenburg, North-East: Stader & Lüneburger land, Lower West: Dümmer & Osnabrücker land, Central East: Börde & Aller lowlands, South-East: Weser hillside & Harz

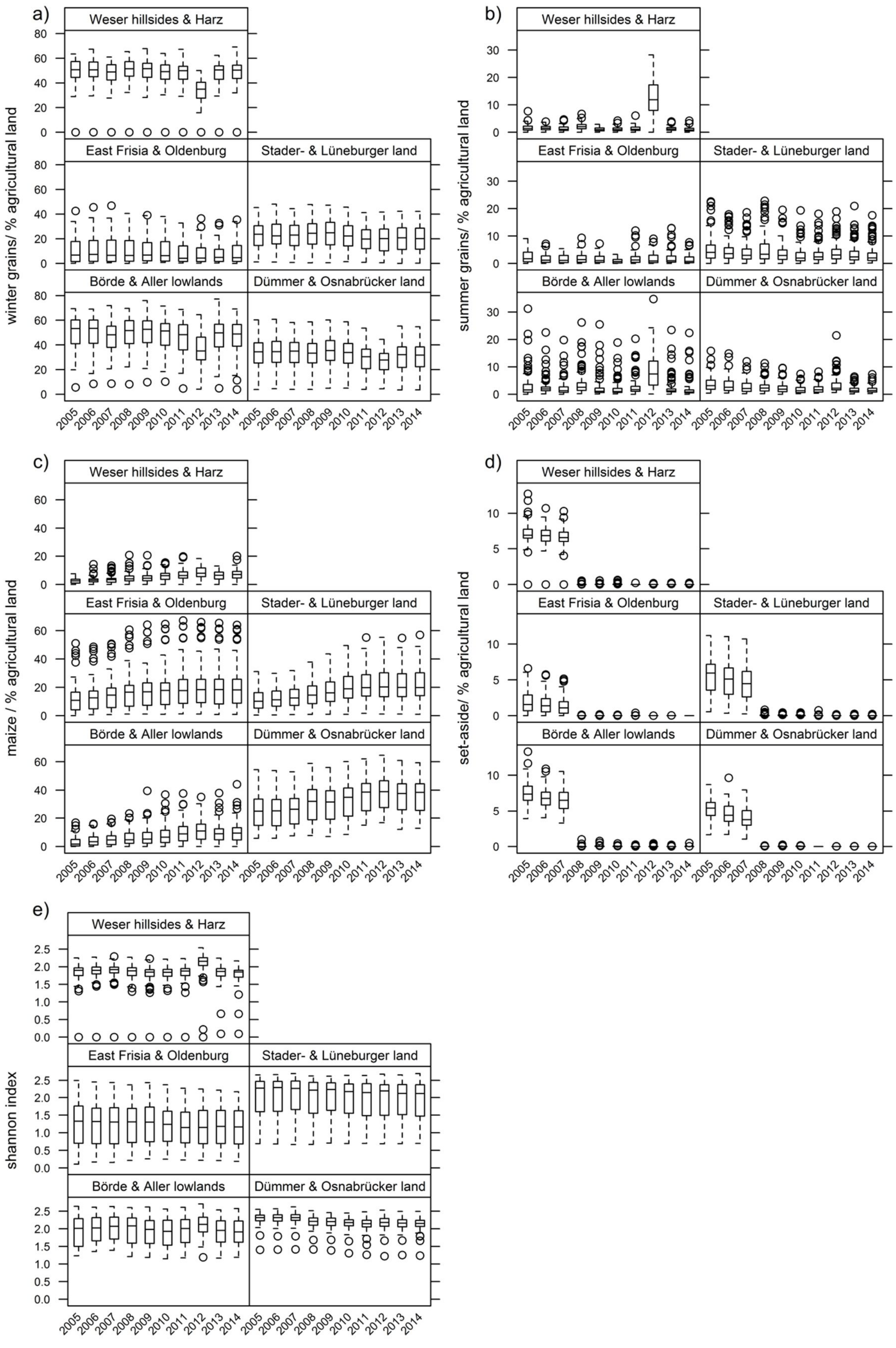

Figure S2: Development of main crop groups over 10 years within 5 different natural regions (Meynen et al. 1962, modified after Strauss) in Lower Saxony. The five regions comprise: North-West: East Frisia and Oldenburg, North-East: Stader & Lüneburger land, Lower West: Dümmer & Osnabrücker land, Central East: Börde & Aller lowlands, South-East: Weser hillside & Harz

Meynen E, Schnmidthüsen J, Gellert J, Neff E, Müller-Miny H, Schultze JH: **Handbuch der Naturräumlichen Gliederung Deutschlands**. Bad Godesberg; 1953-1962.
