# Supplementary material for: Crop diversity loss as primary cause of grey partridge and common pheasant decline in Lower Saxony, Germany
Source: BMC Ecol. 2016 Sep 9;16(1):39. doi: 10.1186/s12898-016-0093-9 (PMC5016946; doi:10.1186/s12898-016-0093-9)
Supplement: Supplementary file 2 — 10.1186/s12898-016-0093-9 List of crops eligible to payments schemes between 2005 and 2014. [file 12898_2016_93_MOESM2_ESM.pdf]

Table S1: List of crops eligible to payments schemes between 2005 and 2014

Cereals

Code	Nomenclature	Groups created for Shannon index	Groups for gam(m)s
113	Durum wheat	durum wheat	summer grain
114	Spelt	spelt	winter grain
115	Common winter wheat (excluding durum wheat)	winter wheat	winter grain
116	Common spring wheat (excluding durum wheat)	spring wheat	summer grain
121	Winter rye	winter rye	winter grain
122	Spring rye	spring rye	summer grain
125	Winter maslin	winter maslin	winter grain
131	Winter barley	winter barley	winter grain
132	Spring barley	spring barley	summer grain
142	Winter oats	winter oats	winter grain
143	Spring oats	spring oats	summer grain
145	Spring maslin	spring maslin	summer grain
155	Triticale	triticale	winter grain
157	Spring triticale	spring triticale	summer grain
171	Grain maize	maize	maize
172	Corn cob mix	maize	maize
174	Sweet corn	maize	maize
175	Companion planting of silage maize and sunflowers	maize other	maize
176[3])[4])	Maize with huntable- or flower strips, which are set aside	maize other	maize
177[3])	Maize with huntable- or flower strips that are farmed with arable crops or harvested	maize other	maize
182	Buckwheat	buckwheat	summer grain
190	All (other) cereals	other	

Protein crop

Code	Nomenclature	Groups created for Shannon index	Groups for gam(m)s
210	Peas for the production of grain	legumes	
220	Beans for the production of grain	legumes	
230	Sweet lupines for the production of grain	legumes	
240	Peas / beans for the production of grain	legumes	
250	Mixtures of peas / cereals	legumes	
290	all (other) pulses for the production of grain	legumes	

Oilseed crops

Code	Nomenclature	Groups created for Shannon index	Groups for gam(m)s
311	Winter rape for the production of grain	winter rape	
312	Spring rape for the production of grain	summer rape	
315	Winter turnip rape for the production of grain	winter Turnip	
316	Spring turnip rape for the production of grain	summer turnip	
320	Sunflowers for the production of grain	oil seed	
330	Soy beans for the production of grain	oil seed	
341	Linseed for the production of grain	oil seed	
342	Fibre flax	oil seed	
390	All (other) oilseeds	oil seed	

Plants harvested green/fodder

Code	Nomenclature	Groups created for Shannon index	Groups for gam(m)s
411	Forage maize (as main fodder)	forage maize	maize
412	Fodder root crops (excluding mangel-wurzel, rutabaga and potatoes)	root crop	
413	Mangel-wurzel	root crop	
414	rutabaga	root crop	
421[1])	Clover	legumes	
422[1])	Clover grass mix	legumes	
423[1])	Lucerne	legumes	
424[1])	Grass as arable silage for stock feed	silage crop	
426	Canary seed (*Phalaris arundinacea*)	silage crop	
427	Other cereals as whole crop silage	silage crop	
428	Rotational grassland	grassland	
429[5])	All other fodder crops from arable land	silage crop	

Permanent grassland

Code	Nomenclature	Groups created for Shannon index	Groups for gam(m)s
441	Reseeding of permanent grassland as substitute for permitted tillage of permanent grassland	grassland	
451	Meadows	grassland	
452	Hay meadows	grassland	
453	Pastures and alps	grassland	
454	Wood pastures	grassland	
462	grazed sandy heaths	external grassland	
463	grazed boggy heaths	external	

		grassland	
464	Grazed nutrient-poor grassland	external grassland	
465	Grazed montane grassland	external grassland	
466	Cut nutrient-poor grassland	external grassland	
467	Cut montane meadows	external grassland	
480	Orchard with grassland farming	external grassland	

Set aside

Code	Nomenclature	Groups created for Shannon index	Groups for gam(m)s
511	Set aside without renewable resources	set aside	set aside
516	Set aside with annual renewable resources	set aside	set aside
517	Set aside with perennial renewable resources	set aside	set aside
545	Set aside according to FELEG GAL	set aside	set aside
555	Set aside for 20 years (arable land)	set aside	set aside
556	Afforestation according to afforestation premium after 1993	afforestation	
558	Set aside for 10 years (arable land)	set aside	set aside
563	Afforestation areas on arable land to activate payment entitlements	afforestation	
564	Afforestation areas on arable land (VO(EG) Nr. 1257/99 or VO(EG) Nr. 1698/2005 as set aside VO(EG) Nr.782/2003 arable land	afforestation	
565	Other set aside sites	set aside	set aside
566	Legumes on set aside	legumes	
567	Afforestation areas on arable land to activate payment entitlements	afforestation	
568	Afforestation areas on arable land without activation of payment entitlements	afforestation	
569	10 year set aside VO(EG) Nr. 1257/99 site without activation of payment entitlements as set aside	set aside	set aside
572	20 year set aside with accompanying measures as set aside without activation of payment entitlements as set aside	set aside	set aside
580	Set aside with growth of renewable resources with declaration for own biogas plant	set aside	set aside

Set aside

Code	Nomenclature	Groups created for Shannon	Groups for gam(m)s

		index	
583[2])	Areas that are not arable but eligible according to Art. 34 (2b (i)) according to VO(EG) Nr. 73/2009 eligible sites	other	
590	Set aside with growth of renewable resources with growth and supply agreement for external biogas plant	set aside	set aside
591	Arable land, temporarily set aside	set aside	set aside
592	Permanent grassland, temporarily set aside	external grassland	

Root crops

**Code**	**Nomenclature**	**Groups created for Shannon index**	**Groups for gam(m)s**
611	Early potatoes	potatoes	
612	Other potatoes / medium and late season potatoes	potatoes	
613	Industrial potatoes, starch potatoes	potatoes	
614	Fodder potatoes	potatoes	
615	Seed potatoes	potatoes	
619	Other potatoes not for starch production	potatoes	
620	Sugar beet	sugar beet	
640	potatoes for starch production; contractors for Südstärke	potatoes	
641	potatoes for starch production; contractors for Emslandstärke	potatoes	
642	potatoes for starch production; contractors for Avebe/D	potatoes	
643	potatoes for starch production; contractors for Avebe/NL	potatoes	
644	potatoes for starch production; contractors for Agrana	potatoes	
690	All other root crops (excluding fodder root crops)	root crop	

Vegetables and other industrial crops

**Code**	**Nomenclature**	**Groups created for Shannon index**	**Groups for gam(m)s**
710	Field grown vegetables	horticulture	
711	Field grown fresh peas	horticulture	
712	Field grown cauliflowers	horticulture	
713	Field grown tomatoes	horticulture	
715	Asparagus	horticulture	

722	Flowers and non-woody ornamental plants (field grown, including wildflowers)	horticulture	
723	Strawberries (field grown)	horticulture	
731	Vegetables and mushrooms under glass or high accessible cover	green house	
732	Flowers and non-woody ornamental plants under glass or high accessible cover	green house	
733	Mushroom beds and vegetal sites in buildings other than greenhouses	green house	
750	Hops	horticulture	
761	Tobacco of group I FLUE CURED (e. g. Virgin D and hybrids …)	other	
762	Tobacco of group II LIGHT AIR CURED (e. g. "Badischer Burley" and hybrids …)	other	
763	Tobacco of group III DARK AIR CURED (e. g. "Badischer Geudertheimer" and hybrids …)	other	
770	Medicinal, fragrant and spice plants	other	
771	Herbs	other	
777	Artichoke	horticulture	
790	All other industrial crops (excluding permanent crops)	other	
791	Horticulture seeds (ornamental plants)	horticulture seeds	
792	Horticulture seeds (fruit and vegetable)	horticulture seeds	
793	Hemp	horticulture	

Perennial and permanent crops

Code	Nomenclature	Groups created for Shannon index	Groups for gam(m)s
811	Drupes and pomes	orchards	
812	Orchards without meadow utilisation	orchards	
815	Pomes e. g. apples, pears	orchards	
816	Drupes e. g. cherries, plums	orchards	
817	Soft fruit e. g. currants, gooseberries, raspberries	orchards	
818	Common sea-buckthorn	orchards	
819	Other orchards e. g. elderberry	orchards	
824	Hazelnuts	nurseries	
825	Walnuts	nurseries	
830	Nurseries, excluding for soft fruit	nurseries	
831	Soft fruit for propagation (in nurseries)	nurseries	
845	Common osier	nurseries	
846	Nurseries of ornamental trees e.g. Christmas tree cultivation	nurseries	

847	Fast-growing wood not short rotation coppice	nurseries	
848[6])	Short rotation coppice	nurseries	
850	Vineyards	nurseries	
890	Other permanent crops	nurseries	
891	Poplars	nurseries	
892	Rhubarb	horticulture	
896	Chinese silver grass (Miscanthus sinensis)	silage crop	
897	Plants for energy production if no other code is available e.g. *Agropyron elongatum*	silage crop	

Other areas

Code	Nomenclature	Groups created for Shannon index	Groups for gam(m)s
910	Other utilised agricultural areas (e.g. browsing areas for wildlife)	flower strips	
912	Propagation of grass seeds	horticulture seeds	
915	Flower strips and field borders (NAU – A5)	flower strips	
918	Perennial flower strips (NAU – A6)	flower strips	
920	home and market gardens	horticulture	
923	grassland without agricultural use	grassland	
924	Biotopes without agricultural use	other	
925	Biotopes with agricultural use	other	
926	trench allotment cooperative wetland	grassland	
930	Utilized water bodies and ponds	water bodies	
940	all other water bodies not utilized	water bodies	
941	Green manure as main crop cultivation		
953	Afforestation areas on arable land VO(EG) Nr. 1257/99	afforestation	
955	Afforestation areas on arable land VO(EG) Nr. 1 257/99 or VO(EG) Nr. 1698/2005 without activation of payment entitlements	afforestation	
960	Dams and dikes	external grassland	
965	Uncultivated peatlands	moorland	
966	Uncultivated heathlands	moorland	
990	All other areas	other	
991	Farm building and track area	other	
992	Mining land, barrens, interfile land, ---, ecological succession areas – permanently set aside	other	
994	Unhitched pits, straw, fodder and dung deposits	other	
995	Forestry area	other	
996	Unhitched pits, straw, fodder and dung	other	

	deposits		
997	Flower gardens, parks, recreation areas, golf courses	other	
998	forests for soil conservation	other	
999	Due to hardship temporarily not utilized areas	other	
