# Supplementary material for: Crop diversity loss as primary cause of grey partridge and common pheasant decline in Lower Saxony, Germany
Source: BMC Ecol. 2016 Sep 9;16(1):39. doi: 10.1186/s12898-016-0093-9 (PMC5016946; doi:10.1186/s12898-016-0093-9)
Supplement: Supplementary file 3 — 10.1186/s12898-016-0093-9 Process of model selection for the Habitat (GAMMs) and Trend (GAMs). [file 12898_2016_93_MOESM3_ESM.pdf]

IV: Process of model selection for the Habitat (GAMMs) and Trend (GAMs) models

Method: Starting from the full model, shown in the formula as r code, all smooth terms were removed and AIC values were compared. The model with the lowest AIC-value was taken as new model to remove smooth terms until the removal of terms could not reduce AIC values anymore. The final model is marked in fat. Latitude* longitude and year were not removed for reasons of spatial and temporal autocorrelation.

Table S2: Model selection for the grey partridge habitat model

formula	Model	df	AIC	BIC	logLik
gamm(log1p(grey partridge breeding pairs)~s(maize)+ s(winter grains) +s(forest) +s(water) + s(summer grains) +s(Shannon) +s(FBm) +s(set aside)+ factor(year) +s(Long, Lat)+, correlation=corAR1(form=~year|municipality), random=list(municipality=~1), method="ML")					
full model	1	34	-5098.64	-4885.71	2583.32
- maize	2	30	-5106.52	-4918.63	2583.26
- winter grains	3	30	-5102.12	-4914.23	2581.06
- forest	4	30	-5078.08	-4890.20	2569.04
- water	5			conversion error	
- summer grains	6	30	-5106.45	-4918.56	2583.22
- Shannon	7	30	-5099.66	-4911.77	2579.83
- FBm	8			conversion error	
- set aside	9			conversion error	
gamm(log1p(grey partridge breeding pairs)~s(winter grains) +s(forest) +s(water) + s(summer grains) +s(Shannon) +s(FBm) +s(set aside)+ factor(year) +s(Long, Lat)+, correlation=corAR1(form=~year|municipality), random=list(municipality=~1), method="ML")					
- winter grains	10	28	-5102.97	-4927.61	2579.48
- forest	11			conversion error	
- water	12	28	-5097.99	-4922.63	2577.00
- summer grains	13			conversion error	
- Shannon	14	28	-5101.22	-4925.86	2578.61
- FBm	15	28	-5105.42	-4930.06	2580.71
- set aside	16	28	-5109.60	-4934.24	2582.80
gamm(log1p(grey partridge breeding pairs)~s(winter grains) +s(forest) +s(water) + s(summer grains) +s(Shannon) +s(FBm) factor(year) +s(Long, Lat)+, correlation=corAR1(form=~year|municipality), random=list(municipality=~1), method="ML")					
- winter grains	17	26	-5104.71	-4941.87	2578.35
- forest	18	26	-5084.19	-4921.35	2568.09
- water	19	26	-5100.81	-4937.97	2576.40
**- summer grains**	**20**	**26**	**-5112.70**	**-4949.87**	**2582.35**
- Shannon	21	26	-5101.77	-4938.93	2576.88
- Fbm	22	26	-5108.70	-4945.87	2580.35
gamm(log1p(grey partridge breeding pairs)~s(winter grains) +s(forest) +s(water) + s(Shannon) +s(FBm) + factor(year) +s(Long, Lat)+, correlation=corAR1(form=~year|municipality), random=list(municipality=~1), method="ML")					
- winter grains	23	24	-5106.94	-4956.63	2577.47
- forest	24	24	-5087.62	-4937.31	2567.81
- water	25	24	-5104.23	-4953.92	2576.11
- Shannon	26	24	-5101.53	-4951.23	2574.77
- FBm	27	24	-5111.93	-4961.62	2579.97

Table S3: Model selection for the pheasant habitat model

formula	model	df	AIC	BIC	logLik
gamm(log1p(pheasant hens)~s(maize)+ s(winter grains) +s(LE) +s(forest) +s(water) + s(summer grains) +s(Shannon) +s(FBm) +s(set aside)+ factor(year) +s(Long, Lat)+, correlation=corAR1(form=~year|municipality), random=list(municipality=~1), method="ML")					
full model	1	32	-883.77	-690.51	473.89
-maize			conversion error		
-winter grains			conversion error		
-LE	2	30	-884.77	-703.59	472.39
- forest			conversion error		
- water	3	30	-880.93	-699.74	470.46
- summer grains	4	30	-887.02	-705.83	473.51
- Shannon	5	30	-872.81	-691.63	466.41
- Fbm	6	30	-882.56	-701.37	471.28
- set aside	7	30	-886.75	-705.56	473.37
gamm(log1p(pheasant hens)~s(maize)+ s(winter grains) +s(LE) +s(forest) +s(water) + s(Shannon) +s(FBm) +s(set aside)+ factor(year) +s(Long, Lat)+, correlation=corAR1(form=~year|municipality), random=list(municipality=~1), method="ML")					
-maize	8	28	-874.92	-705.81	465.46
-winter grains	9	28	-879.96	-710.86	467.98
-LE	10	28	-887.78	-718.68	471.89
- forest	11	28	-823.44	-654.33	439.72
- water	12	28	-884.11	-715.01	470.06
- Shannon	13	28	-875.32	-706.21	465.66
- FBm	14	28	-885.63	-716.53	470.82
-set aside	15	28	-889.68	-720.58	472.84
gamm(log1p(pheasant hens)~s(maize)+ s(winter grains) +s(LE) +s(forest) +s(water) + s(Shannon) +s(FBm) + factor(year) +s(Long, Lat)+, correlation=corAR1(form=~year|municipality), random=list(municipality=~1), method="ML")					
-maize	16	26	-876.81	-719.78	464.40
-winter grains	17	26	-873.56	-716.54	462.78
-LE	18	26	-887.86	-730.84	469.93
- forest	19	26	-826.80	-669.78	439.40
- water	20	26	-886.51	-729.48	469.25
- Shannon	21	26	-879.26	-722.23	465.63
**- FBm**	**22**	**26**	**-890.62**	**-733.59**	**471.31**
gamm(log1p(pheasant hens)~s(maize)+ s(winter grains) +s(LE) +s(forest) +s(water) + s(Shannon) +s(FBm) + factor(year) +s(Long, Lat)+, correlation=corAR1(form=~year|municipality), random=list(municipality=~1), method="ML")					
-maize	23	24	-877.55	-732.60	462.77
-winter grains	24	24	-875.65	-730.70	461.82
-LE	25	24	-886.30	-741.36	467.15
- forest	26	24	-826.24	-681.29	437.12
- water	27	24	-887.20	-742.26	467.60
- Shannon	28	24	-879.03	-734.08	463.52

Table S4: Model selection for the grey partridge trend model

formula	df	AIC
gam(asin(rho grey partride breeding pairs)~s(rho winter grains) +s(rho summer grains) +s(rho maize)+s(rho mean size) + s(rho Shannon) +s(rho set aside)+ s(Long, Lat), method="ML")		
full model	19.485	610.212
-winter grains	19.037	609.112
- summer grains	18.957	610.744
-maize	18.533	608.269
- mean size	18.219	608.745
- Shannon	19.644	612.061
- set aside	18.559	608.214
gam(asin(rho grey partride breeding pairs)~s(rho winter grains) +s(rho summer grains) +s(rho maize)+s(rho mean size) + s(rho Shannon) +s(Long, Lat), method="ML")		
-winter grains	18.098	607.114
- summer grains	17.567	608.309
-maize	17.605	606.268
- mean size	17.691	607.316
- Shannon	18.742	610.076
gam(asin(rho grey partride breeding pairs)~s(rho winter grains) +s(rho summer grains) +s(rho mean size) + s(rho Shannon) +s(Long, Lat), method="ML")		
-winter grains	17.653	605.746
- summer grains	17.156	606.901
- mean size	16.310	604.802
- Shannon	17.777	608.228
gam(asin(rho grey partride breeding pairs)~s(rho winter grains) +s(rho summer grains) s(rho Shannon) + s(Long, Lat), method="ML")		
-winter grains	16.881	605.930
- summer grains	16.316	604.282
- Shannon	18.016	607.646
gam(asin(rho grey partride breeding pairs)~s(rho winter grains) +s(rho Shannon) + s(Long, Lat), method="ML")		
**-winter grains**	**15.529**	**604.143**
- Shannon	16.606	606.089
gam(asin(rho grey partride breeding pairs)~s(rho Shannon) +s(Long,Lat), method="ML")		
- Shannon	16.822	609.512

Table S5: Model selection for the pheasant trend model

formula	df	AIC
gam(asin(rho pheasant hens)~s(rho winter grains) +s(rho summer grains) +s(rho maize)+s(rho mean size) + s(rho Shannon) +s(rho set aside)+ s(Long, Lat), method="ML")		
full model	33.222	560.179
-winter grains	34.531	564.783
- summer grains	34.727	557.871
-maize	26.427	559.528
- mean size	31.971	557.924
- Shannon	26.740	566.708
- set aside	32.507	558.602
gam(asin(rho pheasant hens)~s(rho winter grains) +s(rho maize)+s(rho mean size) + s(rho Shannon) +s(rho set aside)+ s(Long, Lat), method="ML")		
-winter grains	33.583	562.848
-maize	24.972	557.378
- mean size	24.930	557.374
- Shannon	27.006	565.305
- set aside	31.756	556.430
gam(asin(rho pheasant hens)~s(rho winter grains) +s(rho maize)+s(rho mean size) + s(rho Shannon) + s(Long, Lat), method="ML")		
-winter grains	26.720	564.482
-maize	24.425	555.678
- Shannon	19.628	564.597
- mean size	24.466	556.029
gam(asin(rho pheasant hens)~s(rho winter grains) +s(rho mean size) + s(rho Shannon) +s(Long, Lat), method="ML")		
-winter grains	25.899	562.663
- Shannon	18.587	562.843
**- mean size**	**23.526**	**554.117**
gam(asin(rho pheasant hens)~s(rho winter grains) +s(rho Shannon) +s(Long, Lat), method="ML")		
-winter grains	22.788	562.708
- Shannon	17.321	561.632
